# Spectrum of Diseases among Neurosurgical Patients in a Tertiary Care Hospital of Nepal: A Descriptive Cross-sectional Study

**DOI:** 10.31729/jnma.5857

**Published:** 2021-02-28

**Authors:** Harihar Devkota, Nilam Kumar Khadka, Poojan Kumar Rokaya, Pravin Kumar Giri

**Affiliations:** 1 Department of General Surgery, Karnali Academy of Health Sciences, Karnali, Nepal; 2 Department of Neurosurgery, National Academy of Medical Sciences, Kathmandu, Nepal; 3 Department of Orthopedics, Karnali Academy of Health Sciences, Karnali, Nepal; 4 Department of Anesthesia and Critical Care, Karnali Academy of Health Sciences, Karnali, Nepal

**Keywords:** *contusion*, *extradural hemorrhage*, *neurosurgery*, *rural*, *skull fracture*, *traumatic brain injury*

## Abstract

**Introduction:**

The number of neurosurgical cases, especially traumatic injuries, are increasing in remote settings. This study aims to determine neurosurgical cases in a tertiary care center teaching hospital situated in a remote area of Nepal.

**Methods:**

It was a descriptive cross-sectional study among 138 neurosurgical patients at Karnali Academy of Health Sciences from 2019 August to 2020 July. A convenient sampling technique was used. The demographic data, the diagnosis, and management offered were recorded, reviewed, and analyzed using Statistical Package for Social Sciences version 16 and Microsoft Excel.

**Results:**

Out of 138 cases, trauma was the main finding in 102 (73.91%) cases, and fall injury was the most frequent mechanism of injury in 64 (46.38%) cases. Traumatic Brain Injury was the most common disease in 85 (61.6%) cases followed by Prolapsed Intervertebral Disc in 11 (8%), Spine fracture/dislocation in 11 (8%), stroke in 7 (5.1%), spondylosis in 6 (4.3%) and so on. Among the Traumatic Brain Injuries, normal findings were noted in 28 (20.3%) cases, Extra Dural Hemorrhage in 12 (8.7%), contusion 11 (8%), and skull base fracture in 7 (5.1%). With a mean age of 29.8 years, a maximum number of the cases were 30 (21.74%) from the age group 31-40.

**Conclusions:**

Trauma was seen in a majority of neurosurgical cases. Hematoma, Depressed skull fracture, and spinal fractures were the main findings of neurosurgical cases. The burden of neurosurgical cases in this part of the world is quite high, so necessary step should be taken to increase such services.

## INTRODUCTION

Harvey Cushing is considered as the father of neurosurgery, and neurosurgery was established in Nepal in 1989 AD, pioneered by Dr. Upendra Devkota.^1,2^ Neurosurgical cases, especially trauma, are the most frequent cause of morbidity and mortality in populations under 40-45 years of age.^3^–^5^ About two billion people worldwide lack access to even basic surgical services.^6^ About 90% of Nepal people reside in rural areas, and 40% are below poverty.^7^ Most of the patients here have to travel long distances from rural to urban areas where the tertiary level services are centered.^8^

The diagnosis of neurosurgical cases ranges from benign to malignant. The traumatic brain injury, which is the most common presentation, comprises contusion, Diffuse Axonal Injury (DAI), injury from mass lesions, i.e., Hematomas, etc.^9,10^

The study aimed to find out the spectrum of diseases among neurosurgical patients in Karnali Academy of Health Sciences.

## METHODS

This is a descriptive cross-sectional study carried out at Karnali Academy of Health Sciences (KAHS), Teaching Hospital, during one year period from 2019 August to 2020 July. Ethical approval was taken from Institutional Review Committee (IRC KAHS Reference; 25/076/077) for the study.

The sample size was calculated using the formula as follows:

Sample size calculation:


$$\begin{array}{ll} \text{n} & ={\text{Z}}^{\text{2}}\times \text{p}\times \text{q}/{\text{e}}^{\text{2}}\\ & ={\left(1.96\right)}^{2}\times 0.5\times 0.5/{\left(0.09\right)}^{\text{2}}\\ & =118 \end{array}$$

Where,

n = number of sample size
Z = 1.96 at 95% Confidence Interval
p = prevalence, 0.5
q = 1-p
e = margin of error, 9%

The total sample size was calculated as 118. However, 138 patients were included in the study.

A convenient sampling technique was used. The data were traced from hospital records from OPD, Emergency, Ward, OT, and ICU. Care was taken to avoid duplications. The hospital records, as well as the operation notes and radiological findings, were reviewed. Patients' socio-demographic profile, presence of trauma, mode of injury, utilization of types of hospital services, diagnosis, management, and outcome were recorded in standard performa. All patients who received neurosurgical services during the study period were included in the study. The follow-up cases and the cases with incomplete data were excluded from the study.

The collected data were then entered into Microsoft excel and transferred into Statistical Package for Social Sciences (SPSS Inc, Chicago, Illinois) version 16. Data analysis was done, and results were put in tables and figures.

## RESULTS

**Figure 1.**
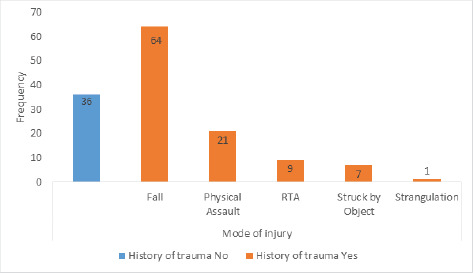
History of trauma and mode of injury.

A total of 138 cases that received neurosurgical services during the study period fulfilled the inclusion criteria and were included in the study cases. There was a history of trauma in 102 (73.91%) cases, while in 36 (26.09%) cases, it was absent. Among the traumatic cases, the fall injuries were 64 (46.36%), physical assaults were 21 (15.22%), Road Traffic Accidents (RTA) were 9 (6.52%), struck by objects were 7 (5.07%), and strangulation was 1 (0.72%) (Figure 1).

Among the 138 patients majority were diagnosed with Traumatic Brain Injury, 85 (61.6%) followed by PIVD 11 (8%), Spine fracture/dislocation 11 (8%), stroke, spondylosis and so on (Table 1).

**Table 1:** Distribution of the diseases according to the diagnosis.

Diagnosis	Frequency n (%)
Traumatic Brain Injury (TBI)	85 (61.6)
Prolapsed Inter Vertebral Disc (PIVD)	11 (8.0)
Spine fracture/dislocation	11 (8.0)
Stroke	7 (5.1)
Spondylosis	6 (4.3)
Scalp injury	6 (4.3)
Seizure disorder	3 (2.2)
Migraine	2 (1.4)
Hypoxic brain injury	2 (1.4)
Trigeminal Neuralgia	1 (0.7)
Osodontoideum	1 (0.7)
Intracranial Space Occupying Lesion (ICSOL)	1 (0.7)
Common peroneal Nerve Injury	1 (0.7)
Arnold Chiari Malformation	1 (0.7)
Total	138 (100.0)

Normal findings were noted in 28 (20.3%). EDH 12 (8.7%) was highest among the cases with findings followed by contusion, skull base fracture, EDH with contusion and so on. Mild cases were highest in number, followed by moderately severe and severe cases. The majority of the head injury cases had no other associated injury 50 (36.2%) while Facial injuries 18 (13%) were second in number, which was followed by Soft tissue injury, chest injury, and so on (Table 2).

**Table 2:** Traumatic Brain Injury, its morphology, severity, and associated injuries.

Morphology based on CT scan findings	Frequency n (%)
Normal findings	28 (20.3)
Closed skull fracture	1 (0.7)
Open skull fracture	3 (2.2)
Depressed skull fracture	5 (3.6)
Open depressed skull fracture	2 (1.4)
Base of skull fracture	7 (5.1)
Diffuse Axonal Injury (DAI)	3 (2.2)
Laceration of brain	1 (0.7)
Contusion	11 (8.0)
Extra Dural Hemorrhage (EDH)	12 (8.7)
EDH, Contusion	6 (4.3)
Sub Dural Hemorrhage (SDH)	1 (0.7)
Sub Arachnoid Hemorrhage (SAH)	1 (0.7)
Intra Cerebral Hemorrhage (ICH)	4 (2.9)
SEVERITY (Glasgow Coma Scale)	
Mild (0-8)	57 (41.3)
Moderate (9-12)	15 (10.9)
Severe (13-15)	13 (9.4)
ASSOCIATED INJURY	
None	50 (36.2)
Soft tissue injury	8 (5.8)
Facial injury	18 (13.0)
Chest injury	4 (2.9)
Limb injury	3 (2.2)
Tympanic membrane rupture	1 (0.7)
Polytrauma	1 (0.7)

**Figure 2.**
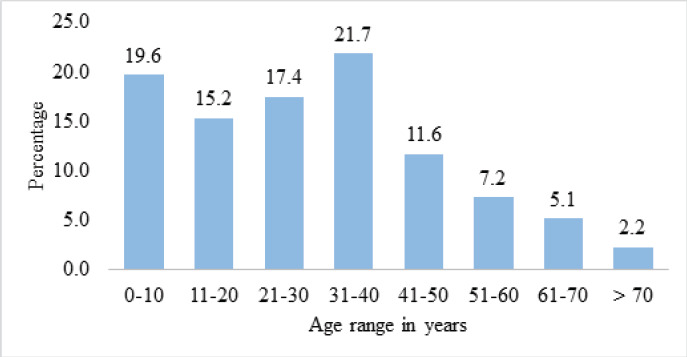
Right superior parathyroid gland at 11 o’clock position in relation to ZT.

Among the patients, 105 (76.1%) were male and 33 (23.9%) were female. The age range was from three months to 76 years, with a mean age of 29.8 and median age of 30 years. A maximum number of the cases were 30 (21.74%) from the age group 31-40, followed by 27 (19.57%) from 0-10, 24 (17.39%) from 21-30 age groups and so on. Three (2.17%) cases were above 70 years (Figure 2).

The service utilization was 92 (67.7%) from the ward, including ICU, 34 (24.6%) from OPD and 12 (8.7%) from the emergency room (Table 3). The ICU stay was ranged from one day to 45 days, with a mean stay of 7.14 days.

**Table 3:** Distribution according to the utilization of the service.

Services	Frequency n (%)
Outpatient Department (OPD)	34 (24.6)
Emergency	12 (8.7)
Ward and Intensive Care Unit Ward (ICU)	56 (40.6)
ICU	36 (26.1)
Total	138 (100.0)

## DISCUSSION

Karnali Province consists of ten districts (Jumla, Humla, Mugu, Dolpa, Kalikot, Rukum-west, Jajarkot, Salyan and Surkeht), with an area of 24,453 km^2^. The people here are economically deprived and have very little access to health. Karnali Academy of Health Sciences is situated in the Heart of Jumla which serves the people in and around Jumla.^11^

In our study, traumatic cases were present in more than two-thirds of the cases, i.e., 73.91%. Among the traumatic cases, fall injury was 46.36%, followed by Physical assault 15.22% and Road Traffic Accident 6.52%. This was quite different from studies in which RTA was the main mechanism of injury conducted by Abdelgadir et al. (60%%) and Sah SK et al. (49.60%).^12,13^ This can be justified as in our setting because of sloppy topography and lack of excess road fall injury led to higher than RTA.

According to the diagnosis made, Traumatic Brain Injury (61.6%) was the most common finding, followed by PIVD (8%), Spine fracture/dislocation (8%), stroke, spondylosis and so on. Other studies also showed a similar TBI pattern with 59.1%^14^ in one and 67.55%^9^ in another. Among the traumatic brain injuries, apart from the normal findings EDH and Contusion were the highest. Mid head injuries were more than moderate which more than severe head injuries were. Most of the head injuries were not associated with other injuries and many were managed conservatively. Agrawal A et al. also showed similar findings.^15^

In our study, most of the cases were male (76.1%). Males were also more common in studies conducted by Oyemolade et al. (70.4%)^9^ and Srinivas M et al. (73%).^16^ The minimum age was three months, and the maximum age was 76 years. A maximum number of the cases were from the age group 31-40 (21.74%), followed by 0-10 (19.57%), 21-30 (17.39%) and so on. Three cases were above 70 years. In a study by Amit Agrawal et al. in 2012, the result is more in the age group 21-30 (27.7%) and then in 31-40 (21.5%) with ages ranging from < 1 to 98 years.^15^

Of the total cases, 26.1% were admitted to ICU and the total length of stay in the ICU ranged from one to 45 days with a mean stay of 7.14 days. Studies conducted by Acharya SP et al. and Akavipat P et al. showed the average length of stay to be less than our study^5,6^ and 2.36 days, respectively.^17,18^

This study was conducted in a single institution, so it may not be generalized to the whole population.

## CONCLUSIONS

Mainly encountered in neurosurgery were the traumatic cases. Fall injury was the highest noted mechanism of injury. Traumatic brain injury was the top diagnosis made, and intracranial hemorrhage, especially Extra-Dural hemorrhage and Contusions, was the major positive CT scan findings. Our study clearly shows the burden of neurosurgical cases in this part of the world, and necessary steps should be taken to increase such services. Development of formal guidelines and recruiting trained or training local surgical personnel can address the problems in delivering Neurosurgical services.
